# The impact of food availability on tumorigenesis is evolutionarily conserved

**DOI:** 10.1038/s41598-023-46896-1

**Published:** 2023-11-14

**Authors:** Sophie Tissot, Lena Guimard, Jordan Meliani, Justine Boutry, Antoine M. Dujon, Jean-Pascal Capp, Jácint Tökölyi, Peter A. Biro, Christa Beckmann, Laura Fontenille, Nam Do Khoa, Rodrigo Hamede, Benjamin Roche, Beata Ujvari, Aurora M. Nedelcu, Frédéric Thomas

**Affiliations:** 1 CREEC/MIVEGEC, Université de Montpellier, CNRS, IRD, Montpellier, France; 2 School of Life and Environmental Sciences, Deakin University, Waurn Ponds, VIC Australia; 3 Toulouse Biotechnology Institute, University of Toulouse, INSA, CNRS, INRAE, Toulouse, France; 4 MTA-DE “Momentum” Ecology, Evolution and Developmental Biology Research Group, Department of Evolutionary Zoology, University of Debrecen, Debrecen, 4032 Hungary; 5 School of Science, Western Sydney University, Hawkesbury Campus, Locked Bag 1797, Richmond, NSW 2753 Australia; 6 Hawkesbury Institute for the Environment, Western Sydney University, Penrith, NSW Australia; 7 AZELEAD, 377 Rue du Professeur Blayac, 34080 Montpellier, France; 8 School of Natural Sciences, University of Tasmania, Hobart, TAS Australia; 9 Departamento de Etología, Fauna Silvestre y Animales de Laboratorio, Facultad de Medicina Veterinaria y Zootecnia, Universidad Nacional Autónoma de México (UNAM), Mexico City, Mexico; 10 Department of Biology, University of New Brunswick, Fredericton, NB Canada

**Keywords:** Cancer epidemiology, Cancer prevention, Cancer therapy, Climate-change ecology, Ecological epidemiology, Evolutionary ecology, Experimental evolution

## Abstract

The inability to control cell proliferation results in the formation of tumors in many multicellular lineages. Nonetheless, little is known about the extent of conservation of the biological traits and ecological factors that promote or inhibit tumorigenesis across the metazoan tree. Particularly, changes in food availability have been linked to increased cancer incidence in humans, as an outcome of evolutionary mismatch. Here, we apply evolutionary oncology principles to test whether food availability, regardless of the multicellular lineage considered, has an impact on tumorigenesis. We used two phylogenetically unrelated model systems, the cnidarian *Hydra oligactis* and the fish *Danio rerio*, to investigate the impact of resource availability on tumor occurrence and progression. Individuals from healthy and tumor-prone lines were placed on four diets that differed in feeding frequency and quantity. For both models, frequent overfeeding favored tumor emergence, while lean diets appeared more protective. In terms of tumor progression, high food availability promoted it, whereas low resources controlled it, but without having a curative effect. We discuss our results in light of current ideas about the possible conservation of basic processes governing cancer in metazoans (including ancestral life history trade-offs at the cell level) and in the framework of evolutionary medicine.

## Introduction

One of the leading hypotheses of evolutionary oncology is that the increase in human cancer incidence in industrialized countries reflects the mismatch between our adaptations to ancestral environments/lifestyles and the fast-changing existence that humans in modern societies are currently experiencing^1^. Among the long list of lifestyle factors that have been proposed to promote oncogenesis in industrialized populations (e.g., lack of exercise, sun exposure, chronic stress, lack of sleep), one that is often cited is diet. For instance, the westernization of the diet in Asian countries, characterized by an overabundance of readily available high caloric foods, has been accompanied by an increase in cancer rates and/or associated mortality (e.g.^2,3^). The exploration of the oncogenic consequences of diet-related evolutionary mismatches is more than ever a timely topic given that many human populations have recently changed (i.e. since the industrial revolution), or are in the process of changing, their dietary habits^4^. Further, as it is common for wildlife to feed on human waste and consume food that they have not evolved with, the problem of food mismatches generating cancers is also relevant to several wildlife species^5,6^.

An important aspect of dietary evolutionary mismatch, at least for humans in modern societies, is not only the quality but also the quantity of food. Indeed, compared to earlier times, a major dietary change in industrialized populations concerns the food intake per meal and the frequency of meals, which have both increased in many geographical areas^7–9^. The generally accepted evolutionary explanation behind this propensity to overeat in these populations is that contemporary humans have remained adapted to past environments, in which it was advantageous in terms of fitness to constantly seek and consume food, because food was scarce and/or of variable availability over time^10^. The preservation of these habits in current times is now maladaptive in most developed countries due to the abundance and regularity of food resources^11^.

Many health problems (e.g., obesity, cardiovascular disease, diabetes, cancers) are associated with the resulting excessive dietary intake, especially when combined with reduced physical activity relative to our ancestors^12–14^. Not surprisingly, several studies have shown that calorie restriction has a protective effect against cancer, especially when applied at an early stage (^15,16^; for review see^17,18^). While previous data indicate that intermittent fasting might be more important than caloric restriction in preventing prostate cancer growth in SCID mice^19^, recent work on a murine breast cancer model revealed that daily caloric restriction is more potent against primary tumor growth and metastatic burden than periods of fasting and diet composition^20^. In a related vein, Simone et al.^21^ proposed that there are actually three types of diets that can not only prevent cancer but also reduce its progression: calorie restriction without malnutrition, intermittent fasting, and the ketogenic diet. All have the advantage of limiting, among other things, the carbohydrate intake necessary for tumor growth with limited, if any, detriment to healthy cells.

Mechanistically, cancer cells’ vulnerability to food availability is thought to be the result of their inability to switch to the cell maintenance program under nutrient-limited conditions^22^. Instead, cancer cells continue to grow and proliferate and become susceptible to stress-induced damage^23,24^. The ability to switch from a reproductive to a survival mode in stressful environments is a fundamental property of all cells and reflects a basic life history trade-off^25^. If the cancer cells’ vulnerability to food availability, well-known in human and rodent models, reflects this fundamental trade-off between reproduction/proliferation and survival/maintenance, we would expect to observe similar outcomes in other animal systems that develop tumors. Note that although not all tumors are, or have the potential to become, cancer (i.e., malignant), tumor development is a pre-requisite for any oncogenic process. Thus, any negative effect of food availability on tumorigenesis will directly affect the potential for cancer and its progression.

To address the possibility that food availability can also affect tumorigenesis in non-human systems, we used a freshwater invertebrate (*Hydra oligactis*) and a vertebrate (the zebrafish *Danio rerio*) model and investigated if/how food levels and frequency can affect the initiation and progression of spontaneous, transmitted, and experimentally induced tumors. Specifically, the experiments in this study were designed to (i) investigate whether the reported increased sensitivity to starvation of cancer cells in humans is also characteristic of tumoral cells in other animal lineages (and might be partially responsible for the reduced incidence of tumors/cancers in the wild) and (ii) address the potential role of evolutionary mismatch (in terms of increased food availability relative to natural/ancestral systems) in promoting tumorigenesis in non-human animal lineages.

**Figure 1:**
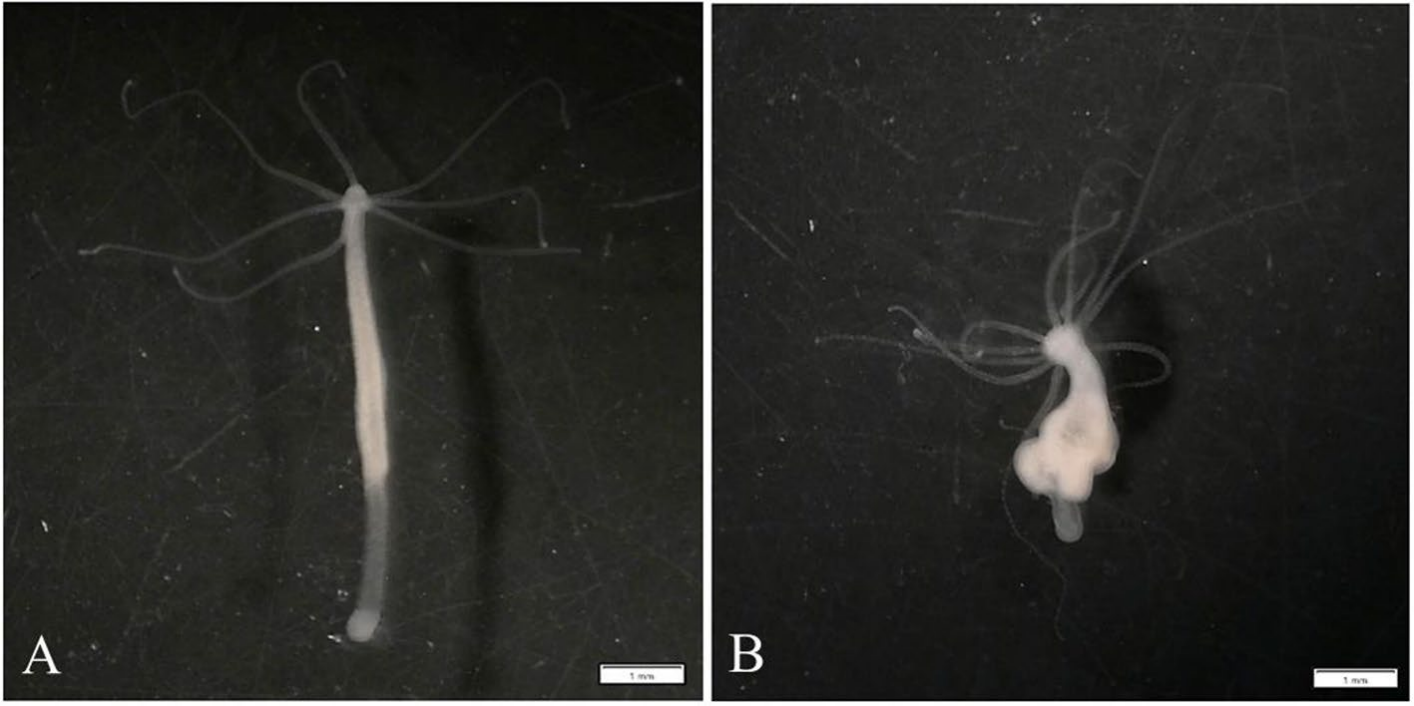
Phenotype of healthy and tumoral hydras from the clonal strains of St. Petersburg. (**A**) Healthy hydra from the tumor-free strain: the body is long and thin, and the number of tentacles does not exceed 7. (**B**) Tumoral hydra from the tumoral strain: many tumefactions thickening the body, and the tentacles are supernumerary (i.e. equal or superior to 8). The pictures are taken with a trinocular magnifier, scale bar: 1 mm.

Both model systems used in this study possess strains that can develop tumors with known dynamics. In hydras, tumor-like abnormal growths (resulting from the uncontrolled proliferation of germline stem cells) have been reported in several species and strains in laboratory settings^26,27^. Such tumors can appear spontaneously, but in some strains, they can also be transmitted vertically during asexual reproduction. Specifically, in one *Hydra oligactis* strain maintained for more than 15 years in the lab, the tumors can be transmitted from the parental polyp to offspring (through the bud), which will develop tumors in about four weeks (see Fig. 1)^26^. In this strain, the tumors cells were shown to differ from normal germline cells in their expression profile, and are characterize by autonomous growth, insensitivity to environmental stimuli and increased invasive capacity^26^. In addition, individuals collected from the wild, which are tumor-free, can sporadically developed tumors when they are kept in the lab^27^. The mechanisms underlying the development and the transmission of these tumors as well as their impact on the biology and the ecology of the hosts are not well understood^28,29^. Notably, at least in one *H. oligactis* strain bearing transmissible tumors, the tumor-associated microbiome might also play a role in the maintenance of tumors^30^.

**Figure 2:**
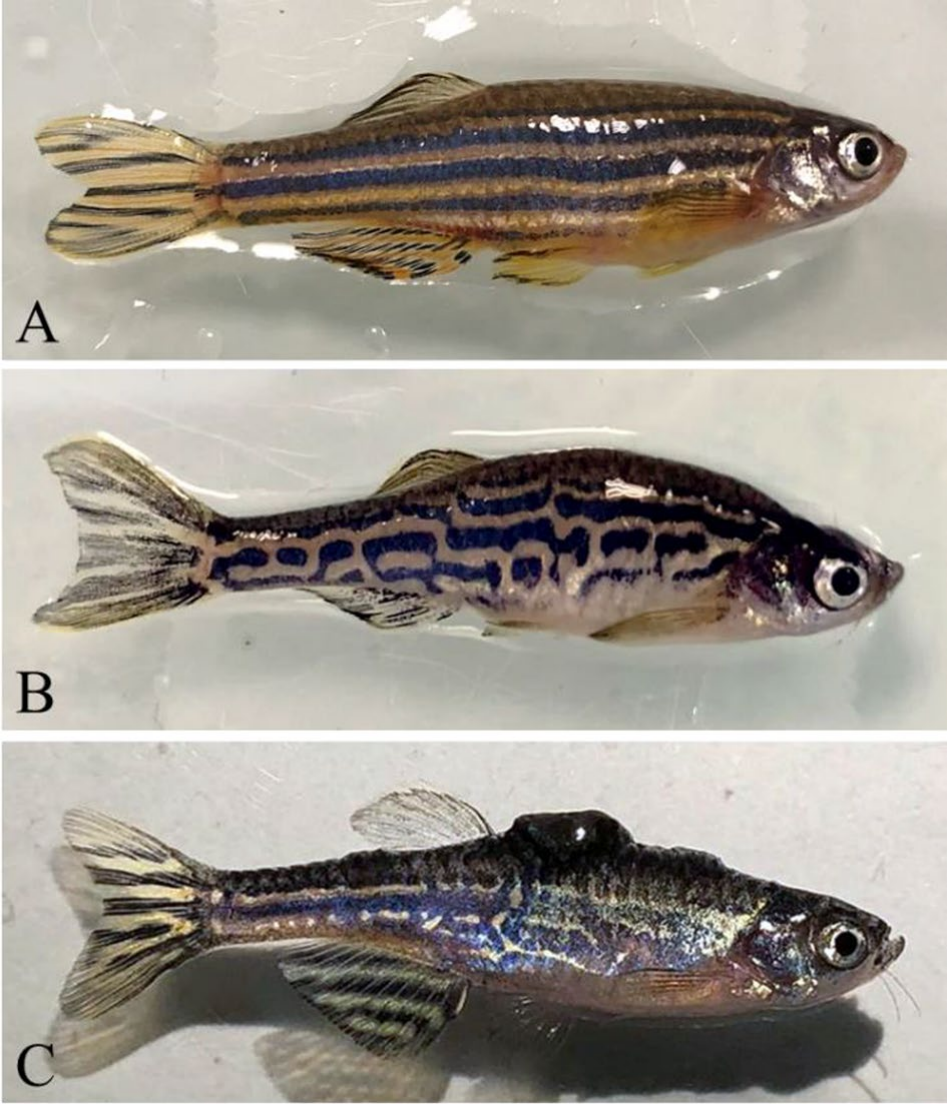
Phenotype of healthy and tumoral zebrafish. (**A**) Healthy zebrafish from the AB strain. (**B**) Zebrafish with nevi (i.e. premalignant lesions) from the kita-GFP-RAS transgenic strain: the pigmentation pattern is altered. (**C**) Zebrafish with nevi and tumor from the kita-GFP-RAS transgenic strain: in addition to the modified pattern, a melanoma has developed on the fish’s back.

In zebrafish, a genetically modified strain has been developed that spontaneously develops a pigmentation abnormality (i.e. nevi) that can then progress into skin tumors in almost 100% of cases (see Fig. 2). In this strain, tumor formation is initiated and maintained by HRAS oncogene expression, resulting in melanomas immunologically similar to human melanomas^31^. We used both healthy individuals as well as tumor-bearing individuals at early and late stages of tumorigenesis (i.e., before and after the appearance of nevi or tumors).

We formulated three hypotheses and specific predictions. (i) Under nutrient deprivation, pre-cancerous cells that are defective in controlling their proliferation are unable to activate their maintenance program and will continue to divide and sustain stress-induced damage and death; thus, low resource levels or fluctuations in resource levels (such as during calorie restriction or intermittent fasting, which are likely to characterize natural systems) should have a preventive or even curative/purging role during early stages of tumorigenesis. (ii) Abundant and/or frequent food availability (reflecting artificial settings; i.e., evolutionary mismatch) allows pre-cancerous cells to avoid the consequences of the proliferation/maintenance trade-off (and its purging role) induced by nutrient limitation and take advantage of available resources to promote proliferation; thus, such diets facilitate tumorigenesis and/or tumor progression. (iii) As tumor cells accumulate genetic and epigenetic disorders, they are less affected by ancestral trade-offs (since they acquire additional ways to withstand stress in response to changes in the tumor microenvironment); thus, fully developed tumors should be less impacted by the scarcity of resources. Additionally, a lack of resources at this late stage may in fact promote tumor progression by lowering the efficiency of host anticancer defenses.

## Materials and methods

**Figure 3:**
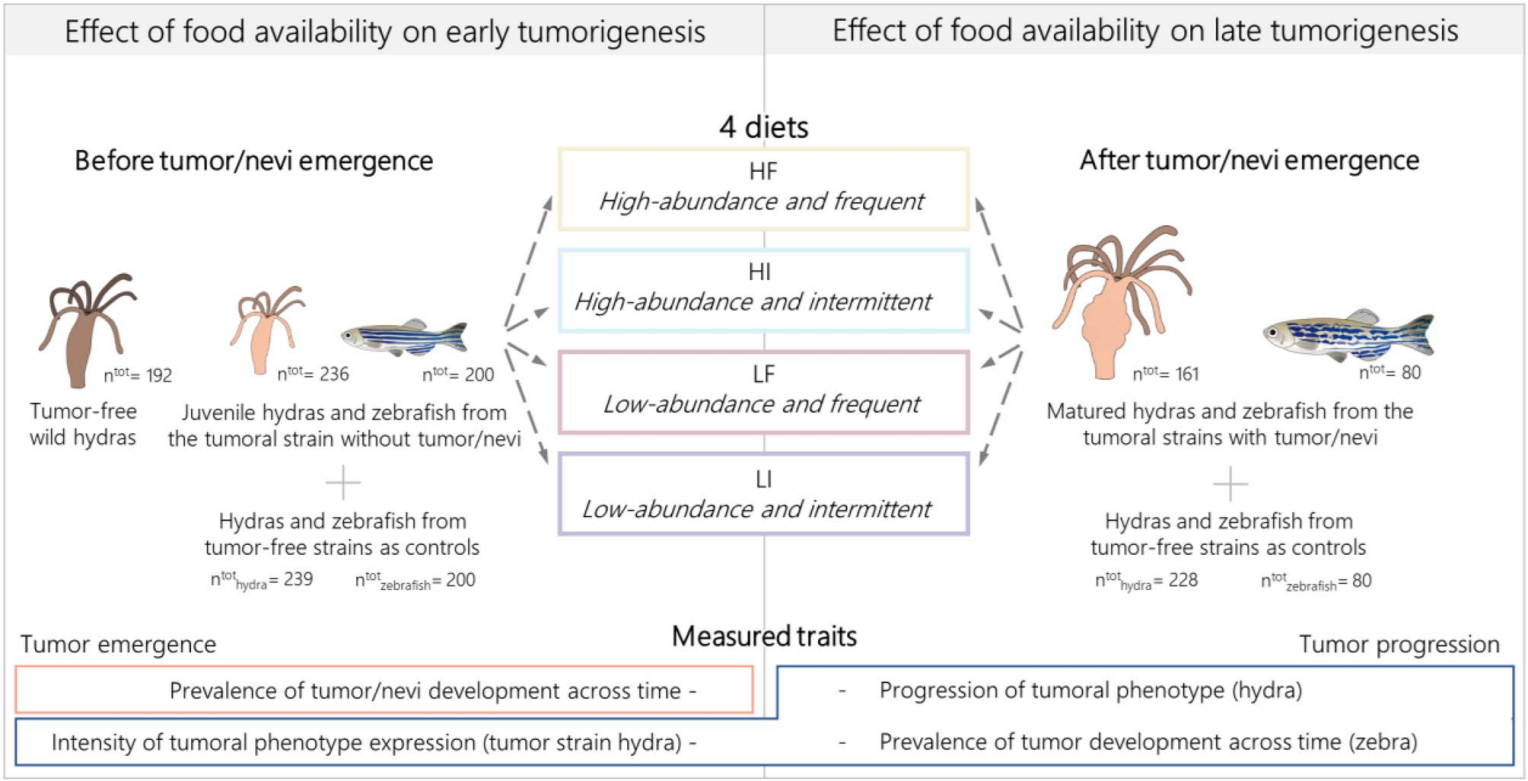
Graphical summary of the general experimental design.

The general experimental design used in this study is summarized in Fig. 3 and detailed in the next sections.

### Hydra

We used two different sets of hydras. One set originated from the so-called St. Petersburg lineage (*H. oligactis*), which includes both healthy (tumor-free) and tumoral clonal strains that have been kept in culture for more than 15 years (described in^26^) (see Fig. 1). The other set was composed of wild hydras collected from the field (Montaud lake in France; 43° 44′ 52″ N; 3° 59′ 23″ E) on May 2nd, 2022; under our lab conditions, these wild hydras can spontaneously develop tumors^32^. The specific lab culture conditions are detailed in^29^; briefly, polyps are grown at 18 °C and fed ad libitum three times a week with artemia nauplii.

To test the effect of nutrition on tumor emergence, we used freshly detached hydras from the tumor-free and the tumoral strain of the St. Petersburg lineages. For each strain, 240 hydras divided in four groups corresponding to the feeding regimes described below, were placed individually in standard cell culture plates (12-well plates, Thermo Scientific, 1.5 ml/well), which were changed every month. Note that at this stage, the tumors are not visible.

To evaluate the effect of nutrition on tumor progression, the experiment was started with individuals that already developed tumors. To obtain those individuals, four groups of 60 juveniles from the tumor-free strain and 60 juveniles from the tumoral strain were placed in similar plates for five weeks, as in the previous experiment. They were fed ad libitum three times a week with artemia nauplii (*Artemia salina*, Planktovie S.A.S., Marseille, France) hatched according to the procedure described in^21^; 8 h after feeding, the remaining artemia were removed from the wells and the water level was restored. The experiment was initiated when the hydras were five weeks old – corresponding to the time when the tumor phenotype emerges in the tumoral strain (i.e. tumors are visible size and the number of tentacles increases^26^). At that time, the tumoral strain presented heterogeneous intensities of expression of the tumor phenotype depending on the individual (discussed later). Details on the number of juveniles and aged hydras at the start of the experiment (i.e. considering the mortality that occurs between birth and the start of the experiment) for each batch, diet and status are available in the supplementary material (Table S1).

Concerning the wild hydras from Montaud, directly after sampling, four groups of 48 individuals were placed individually under the four different feeding regimes in the same plates as described above. Details on the numbers of individuals for each diet are available in the supplementary material (Table S2).

All the polyps were exposed to four diets: (i) high-abundance and frequent, with ad libitum feeding five times a week, (ii) high-abundance and intermittent with ad libitum feeding once a week, (iii) low-abundance and frequent feeding with 5 artemia individuals five times a week, and (iv) low-abundance and intermittent feeding with 5 artemia individuals once a week. For juvenile hydras, the appearance of tumors was monitored for two months, and the intensity of expression of the tumor phenotype was quantified visually, under a dissecting microscope, using a 6-level ordinal scale. The assessment was carried out by two independent experimenters, one of whom was unaware of the regime applied. In 100% of cases, there was consensus between the two experimenters on the score levels. The 6-level scale was validated using the ratio of large to small germinal cells (the cell type associated with tumors in hydras,^26^) ensuring that visual tumor progression matched the accumulation of abnormal cells. For 5-week-old tumoral hydras, the tumor phenotype expression was measured at the beginning of the experiment and one month later. Full detail on the scale used for the measurement is provided in the supplementary material (Figs. S1 and S2).

### Zebrafish

To non-invasively measure the prevalence and the timing of nevi and tumors in zebrafish, we used the kita-GFP-RAS transgenic zebrafish line, which is known to spontaneously develop premalignant lesions (nevi) and tumours/melanomas (see Fig. 2)^31^. The strain AB was used as control. Larval and adult zebrafish were maintained on a light/dark cycle of 12/12 h in a system with a partial renewal of the water up to 30%. Embryos were obtained by natural spawning of breeding pairs and were kept at 28.5 °C in the tank water. At 9 days post fertilization (dpf) the larvae were introduced into the rearing system, with 50 individuals in a tank in order to obtain at least 20 fish at the age of three months. All experimental procedures on zebrafish were carried out in accordance with European directives and French Ministry of Health animal protection regulations (approval number: F341725) by the company Azelead based in Montpellier, France. The experiments were approved by the ethics committee for animal experimentation (Direction départementale de la protection des populations—Hérault). The study was conducted in accordance with ARRIVE guidelines.

Considering the high natural mortality at the larval stage, expected to affect about 50% of the individuals (L. Fontenille, *personal communication*), the number of larvae used to test the effect of nutrition on tumor emergence (i.e., nevi onset) was fixed at 200 larvae from the tumoral strain (i.e. kita-GFP-RAS strain) and 200 larvae from the control strain (i.e. AB strain). For each strain, the larvae were separated into four groups and placed in identical aquariums to those used for rearing. These zebrafish were fed with four diets from 5 dpf.

To evaluate the effect of nutrition on tumor progression, 400 larvae were placed in identical aquariums and fed in a conventional manner (described in supplementary, Table S3) for three months. Then, for the tumoral strain, 80 individuals with nevi were selected and separated into the four experimental groups; for the control strain, 80 individuals were also randomly selected and subjected to the same treatment as the tumoral strain.

All zebrafish were fed with four different diets, using the same categories as for the hydra (see above), but this time on a dry food basis and with artemia as enrichment. For juvenile zebrafish (i.e. 5dpf), the diets have been adapted to follow the change in their food needs over time and respected the differences between each diet. The constitution of the different diets used for zebrafish is available in the supplementary material (Table S3). Juvenile zebrafish were followed up for six and a half months in order to check the appearance of nevi, while three-month-old zebrafish that already had nevi were followed up for seven months to check the appearance of tumors.

### Data analysis

The analyses presented here were performed with the R software (version 4.2.2)^33^ and the graphical representations were realized with the “GGplot2” package^34^. The prevalence of tumors across time for hydras from the tumor-free and the tumoral strains, and of nevi and tumors for zebrafish from the tumoral strain were analyzed according to the diet with survival regressions. These specific regressions allow to treat censored data (which are used when the event of interest was not observed during the experiment) whether or not the instantaneous risk of observing the phenomenon of interest (in this case, the appearance of a tumor or nevi) is constant, by changing the distribution law in the model^35^. The intensity of tumor phenotype expression of hydras from the tumoral strain and its progression over one month were analyzed according to diet with respectively cumulative link models (CLM) and cumulative link mixed models (CLMM). A random effect was included for the latter to account for the lack of independence between repeated measurements of each individual. The number of replicates was too small to quantify potential batch effects in hydra experiments while the zebrafish experimental design only used a single batch. Table 1 summarizes the distribution, function, and associated packages used to explore each trait. Then, the procedure detailed in^36^ was followed to operate the model selection based on the weight of the Akaike information criterion (AIC) for CLM and on its corrected version (AICc) for CLMM. The formulas of all the different models constructed, as well as the value of AIC or AICc’s weight were obtained with the "MuMIn" package^37^, and are detailed in the supplementary material (Table S4). The prevalence of tumors for wild hydras was compared between diets with a test of given proportion.

**Table 1:** Details of distributions, functions, and R packages used for each trait measured for juvenile hydras (*H. oligactis*), juvenile zebrafish (*D. rerio*), five-week-old hydras, and three-month-old zebrafish.

Biological model	Trait	Distributions, functions, and R packages used	
		Juvenile individuals	Mature individuals
Hydras from tumor-free strain	Tumor prevalence	Weibull *survreg* (survival^38^)	
	Tumor timing		
Hydras from tumoral strain	Tumor prevalence	Weibull *survreg* (survival^38^)	
	Tumor timing		
	Tumor size	Logistic *clm* (ordinal^39^)	Logistic *clmm* (ordinal^39^)
Zebrafish from tumoral strain	Nevi prevalence	Weibull *survreg* (survival^38^)	
	Nevi timing		
	Tumor prevalence		Weibull *survreg* (survival^38^)
	Tumor timing		
Wild hydras	Tumor prevalence	Binomial *prop.test* (stat^40^)	

Functions are presented in italics and the associated R packages in parentheses.

For the experiments with hydras, the appearance of spontaneous tumors in individuals from the tumor-free strain was monitored. In contrast to the tumors in the St. Petersburg tumoral strain—which have been vertically transmitted for at least 15 years, these spontaneous tumors are known to appear only sporadically and with a low prevalence (a more detailed description is available in^32^). Therefore, the prevalence of these spontaneous tumors was analyzed according to the diet treatment using a survival regression as previously described in^38^. The formulas of all the different models constructed, as well as the value of AIC or conditional AIC’s weight are detailed in the supplementary material (Table S4) and details of the model selected are summarized in Table 1.

## Results

### The effect of food availability on tumorigenesis in juvenile hydras from the St. Petersburg lineage

To assess the effect of food quantity and frequency on tumor emergence and/or progression, juvenile hydras from both tumor-free and tumoral strains (before tumors are visible) were subjected to four diets varying in food quantity and frequency. For hydras from the tumor-free strain we recorded the rate of appearance of spontaneous tumors. For hydras from the tumoral strain, tumor development was monitored for two months and, at the end, the intensity of tumor phenotype expression was measured to assess the effect of feeding on tumor progression.

**Figure 4:**
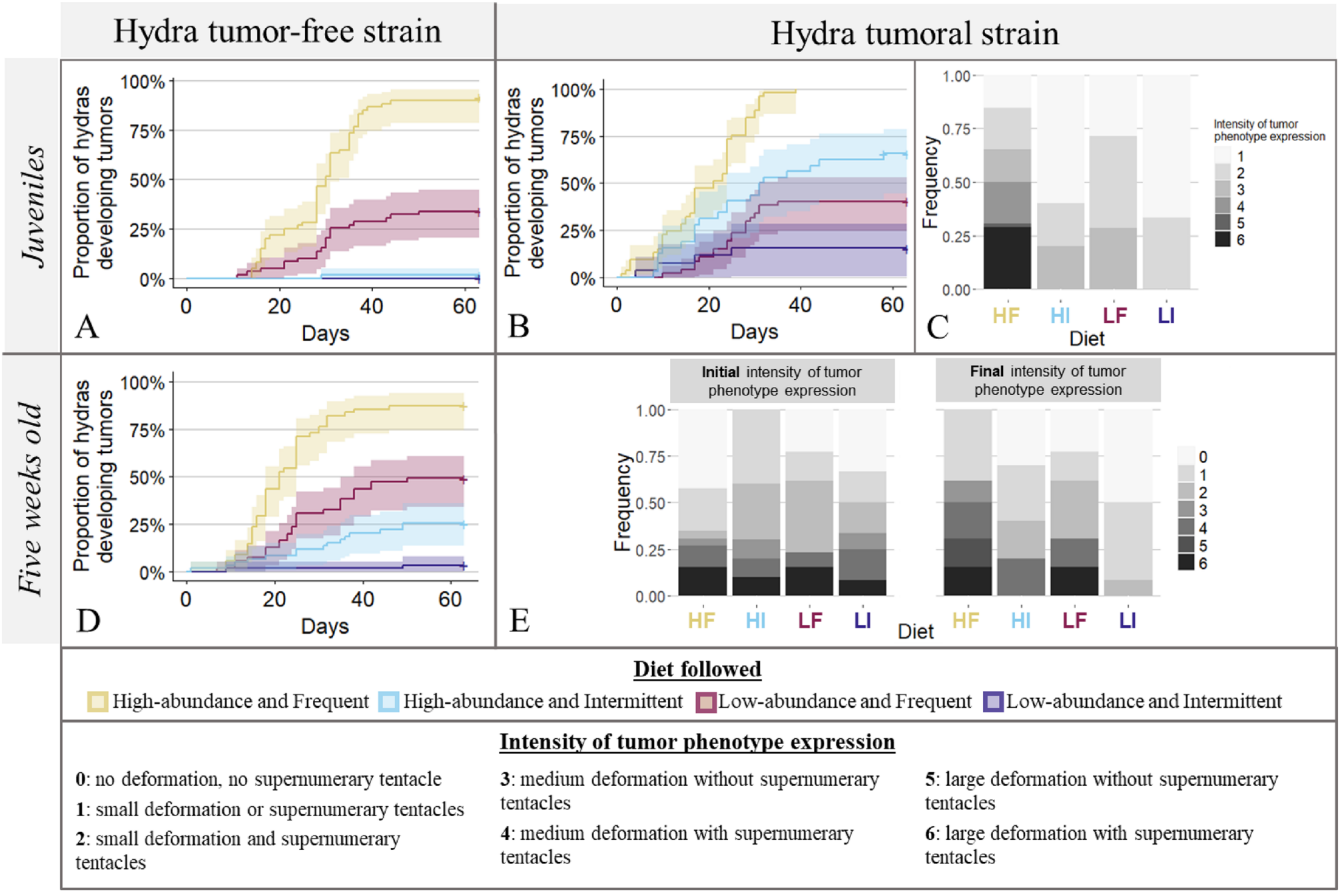
The effects of four diets on tumorigenesis in hydras from tumoral and tumor-free St. Petersburg strains. (**A**) The proportion of juvenile hydras from the tumor-free strain developing spontaneous tumors across time (days) according to the diet followed. (**B**) The proportion of juvenile hydras from the tumoral strain developing tumors across time (days) according to the diet followed. (**C**) The frequency of the intensity of tumor phenotype expression in juvenile hydras from the tumoral strain according to the diet followed. (**D**) The proportion of five-week-old hydras from the tumor-free strain developing spontaneous tumors across time (days) according to the diet followed since their fifth week. (**E**) Changes in the frequency of the intensity of tumor phenotype expression in hydras from the tumoral strain (five-week-old) according to the diet followed over one month (i.e. after tumor development).

We found that diet significantly affected the emergence of spontaneous tumors in juvenile hydras from the tumor-free strain. The proportion of individuals developing tumors increased as the frequency and/or quantity of food increased. The high-abundance and frequent diet led to a significantly higher rate (90% vs. 35%) of tumor occurrence (Fig. 4A; survival regression; estimate = 0.07, SE = 0.04, *p*-value = 1.5e−06) compared to the low-abundance and frequent diet (Fig. 4A; survival regression; estimate = 0.19, SE = 0.53, *p*-value = 2e−03).

For juvenile hydras from the tumoral strain, the models selected to explain the tumor development across time indicated a significant effect of the diet. The high-abundance and frequent diet was associated with the highest tumor development rate (Fig. 4B; survival regression; estimate = 0.11, SE = 0.04, *p*-value = 3.4e−10), followed by the high-abundance and intermittent diet (Fig. 4B; survival regression; estimate = 0.27, SE = 0.10, *p*-value = 3.9e−04), the low-abundance and frequent diet (Fig. 4B; survival regression; estimate = 0.46, SE = 0.17, *p*-value = 3.3e−02), and finally the low-abundance and intermittent diet with the lowest tumor development rate.

Interestingly, in the tumor-free strain, the low-abundance and frequent diet has a stronger effect compared to the high-abundance and intermittent diet whereas we observed the reverse situation in the tumoral strain. Food frequency seems to be more important in the former case, whereas abundance seems to be more important in the latter.

Lastly, for hydras in the tumoral strain that developed tumors, the model selected to explain the intensity of tumor phenotype expression includes the effect of the diet, revealing that hydras on a high-abundance and frequent diet developed more intense tumor phenotypes than those on the other three diets (Fig. 4C. CLM; reference group: high-abundance and intermittent diet, OR = 10.32, SE = 9.66, *p* value = 1.3e−02).

### The effect of food availability on tumorigenesis in five-week-old hydras from the St. Petersburg lineages

Five-week-old hydras from both the tumor-free and the tumoral strains were subjected, as above, to four diets varying in food quantity and frequency. The rate of spontaneous tumor development in hydras from the tumor-free strain was recorded. For hydras from the tumoral strain (with visible tumors), the intensity of tumor phenotype expression was measured at the beginning of the experiment and one month later.

Among the models fitted to explain the proportion of hydras from the tumor-free strain developing spontaneous tumors, the model with the best AIC contained a significant effect of the diet applied at five weeks of age. The proportion of hydras that developed spontaneous tumors increased with the frequency and quantity of provided food. The low-abundance and intermittent diet led to the lowest spontaneous tumor development rate with less than 5% of individuals showing tumors. The high-abundance and intermittent diet presented a higher rate with 25% of individuals developing tumors (Fig. 4D; survival regression; estimate = 0.20 SE = 0.12, *p*-value = 5.05e−03), the low-abundance and frequent diet has a rate of 50% of individuals (Fig. 4D; survival regression; estimate = 0.10, SE = 0.06, *p*-value = 8e−05), and finally the high-abundance and frequent diet with shows the highest rate of 85% of individuals developing spontaneous tumors (Fig. 4D; survival regression; estimate = 0.04, SE = 0.02, *p*-value = 3.6e−08).

In the tumoral hydra strain, we detected a significant effect of the interaction between diet and time on tumor progression. The intensity of tumor phenotype expression increased over time in hydras in the high-abundance and frequent diet group (Fig. 4E; CLMM; OR = 5.00, SE = 3.44, *p*-value = 1.9e−02) while it decreased in hydras in the low-abundance and intermittent diet group (Fig. 4E; CLMM; OR = 0.18, SE = 0.15, *p*-value = 3.8e−02). In addition, we observed a high mortality rate (half-life of less than 30 days) in hydras in the low-abundance and intermittent diet group. For those individuals, the intensity of tumor phenotype expression was measured on moribund animals, whose body was altered (i.e. the body became thinner and tentacles got shorter, then the polyp started to fragment), leading to an underestimation of this intensity.

### The effect of food availability on tumorigenesis in wild hydras

**Figure 5:**
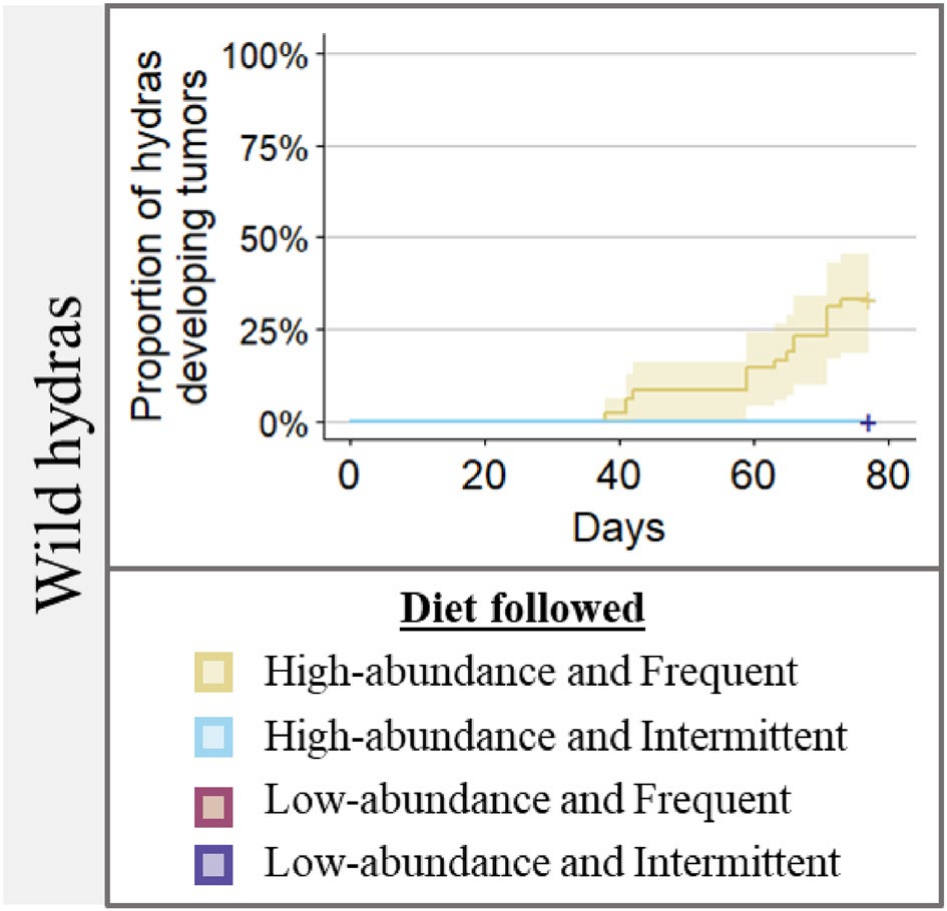
The proportion of wild hydras developing tumors across time (days) according to diet.

In contrast to the tumor-free St. Petersburg strain, we found that wild hydras only developed spontaneous tumors in the high-abundance and frequent food treatment, with a prevalence of only 33% (compared to 90% in the tumor-free St. Petersburg strain) (Fig. 5; test of given proportion; X-squared = 16.87, *p*-value = 3.99e−05).

### The effect of food availability on tumorigenesis in juvenile zebrafish

**Figure 6:**
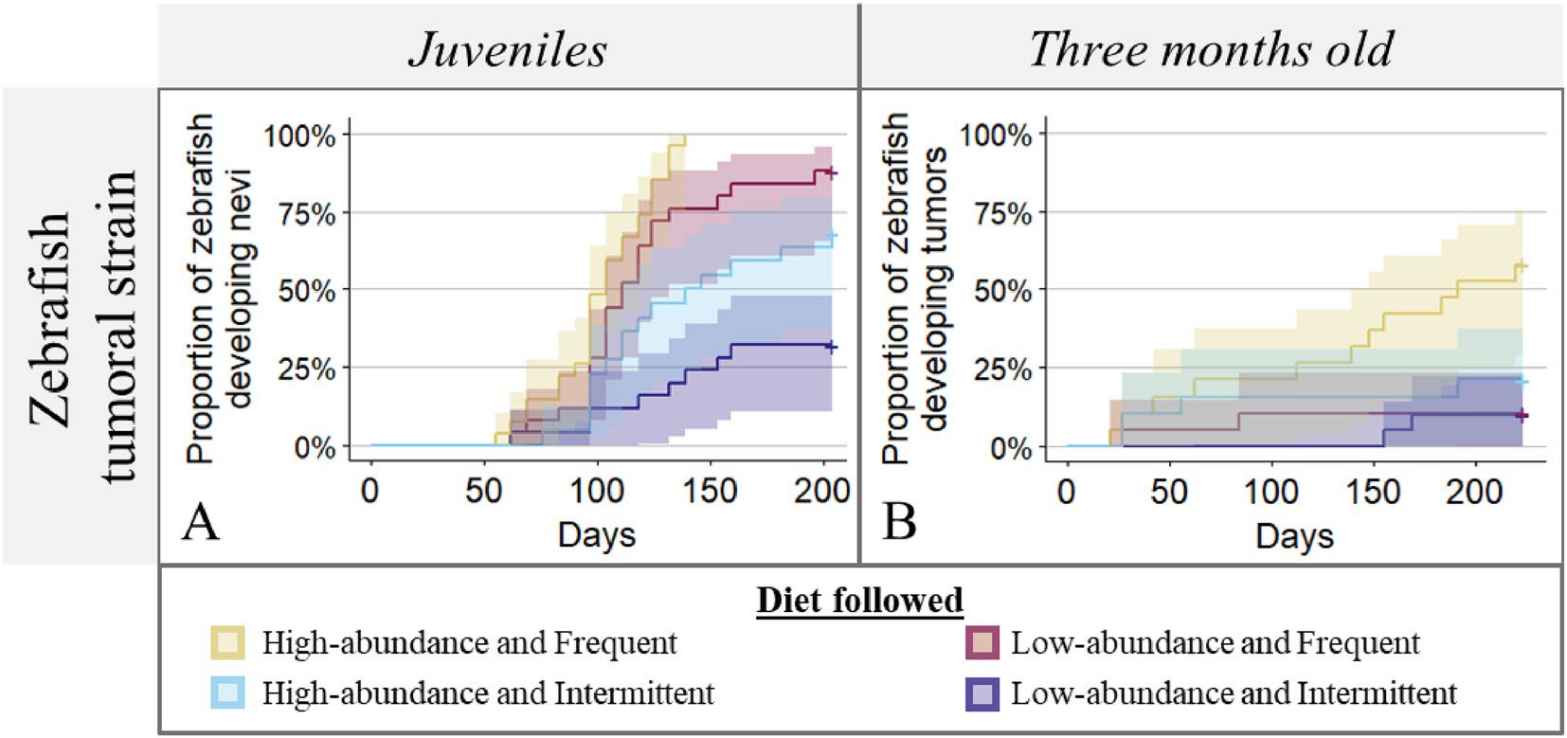
The effects of four diets on tumorigenesis in zebrafish from the tumoral strain. (**A**) The proportion of juvenile zebrafish from the tumoral strain developing nevi across time (days) according to the diet followed. Given the high natural mortality (see “Materials and methods”), the analysis was performed only on individuals still alive at the end of the study or having developed nevi before dying. (**B**) The proportion of zebrafish with nevi from the tumoral strain developing tumors across time (days) according to the diet followed since their third month (i.e. after nevi development).

Juvenile zebrafish from both control and tumoral strains were subjected to four diets varying in food quantity and frequency, and tumor onset was surveyed for 6 months and a half. Zebrafish from the control strain did not show nevi or tumor development, regardless of diet. In the fish from the tumoral strain, the proportion of individuals developing nevi is well explained by the effect of the diet; an increase in food availability was associated with an increased development of tumors. Zebrafish exposed to the high-abundance and frequent diet had the highest nevi development rate with 100% of individuals affected (Fig. 6A; survival regression; estimate = 0.40, SE = 0.05, *p*-value = 3.6e−12), followed by the low-abundance and frequent diet with 88% of individuals affected (Fig. 6A; survival regression; estimate = 0.54, SE = 0.07, *p*-value = 4.7e−06), the high-abundance and intermittent diet with 68% of individuals affected (Fig. 6A; survival regression; estimate = 0.69, SE = 0.10, *p*-value = 9.1e−03), and finally the low-abundance and intermittent diet with 32% of individuals developing nevi.

### The effect of food availability on tumor progression in three-month-old zebrafish

Three-month-old zebrafish from the control strain and tumoral strain (with visible nevi) were also fed the four diets. Tumor development and/or progression (i.e. from nevi to tumors) was studied for two to seven months. Neither nevi nor tumors were observed in the zebrafish control strain in any of the four diet treatments. Concerning the proportion of zebrafish from the tumoral strain developing tumor across time, the relevant model contained the significant effect of diet. Zebrafish following the high-abundance and frequent diet had higher tumor development rates than those in the three other diets, with 55% of individuals affected (Fig. 6B; survival regression; reference group: high-abundance and intermittent diet, estimate = 0.15, SE = 0.12, *p*-value = 1.8e−02).

## Discussion

Extensive research on the roles of diet on human cancer dynamics resulted in important advances over the past decades^41–45^. For example, it is now clearly established that many cancers can be prevented by adopting appropriate dietary measures^46–48^. There is also growing evidence that certain diets are more favorable than others in terms of the likelihood of recovery in cancer patients^49^. However, the subject remains topical, particularly because numerous human populations, especially in industrialized countries, have recently altered, or are in the process of, changing their dietary habits^4^. This will likely generate some evolutionary mismatches with still under-investigated oncological consequences^1^. The objectives of this study were to (i) test whether food quantity and frequency affect tumor initiation and progression in non-human systems, and (ii) identify whether the vulnerability of tumoral cells to nutrient availability is a conserved trait in evolutionarily distant animal lineages (i.e., mammals, fish and hydra).

### The effect of food intake on tumor emergence

In hydra, we explored the link between tumor emergence and diet by considering three categories of polyps. They included two types of healthy polyps and one polyp type presumably tumoral but asymptomatic. The healthy polyps included (i) polyps from the tumor-free St. Petersburg laboratory strain, and (ii) polyps from the wild, which are likely healthy since tumor polyps have never been found in nature^32^. The presumed tumoral polyps were juvenile polyps from the St. Petersburg tumoral strain that just detached from tumoral parents; in standard laboratory conditions, these young polyps usually develop tumors in about four weeks as a result of vertical transmission (see^26^). Our results confirm that diet is a key variable influencing the likelihood of tumor occurrence, with frequent overeating being a risk factor favoring tumor emergence, whereas lean diets (restricted diet and/or intermittent fasting) appear to be protective.

For the two categories of healthy hydras, it is unclear if high food availability is directly responsible for (i.e., induces) the initiation of tumors, and/or if it favors the accumulation of already present abnormal cells that originated due to other reasons. The food we used (artemia) is not itself mutagenic, but its high availability is likely to boost cell proliferation (since the budding rate of hydras is positively correlated with food intake^50–52^) and hence tumoral risks linked to cell divisions. This impact of food abundance is likely due to metabolic effects because reduced calorie intake produces metabolic reprogramming^53^ that can prevent or slow down cancer progression^54^. Of note, recent studies showed that reduced caloric intake and periodic fasting do not have the same metabolic effects. The different effects of these modes of caloric restriction might explain their differential impact on tumor growth and metastatic burden^20^, suggesting a possible explanation for the effects of the different diets in this study. Furthermore, because tumor development in *H. oligactis* is mediated by bacteria (i.e. Rathje et al.^30^ demonstrated that the development of tumors is induced and persists only in the presence of a bacterial conflict between bacteria from the genus *Pseudomonas* and the spirochete *Turneriella*), we cannot rule out that food availability alters the composition of the microbiome, which in turn influences tumorigenesis. In any case, our findings support the hypothesis that low food availability has a protective effect, perhaps by limiting the energy supply and/or raw material for biomass expansion (e.g. carbon, phosphorous, nitrogen) that tumoral cells need to grow and proliferate. Since this observation also applies to hydras from the tumoral strain (which inherited tumoral cells), it suggests that the protective role of lean diets is strong and durable even when tumoral cells are already present inside the body.

It should be noted, however, that although the hydras of the St. Petersburg strains developed fewer tumors when maintained on the three food limiting regimes, they also showed a lower survival rate relative to the hydras in the high-abundance and frequency regimen (see supplementary data). This difference in survival can probably be attributed to the fact that the food limiting regimes we used imposed a long-term malnutrition for this strain. Nevertheless, wild hydras do not show differential mortality between the different diets, suggesting that the increased mortality with decreased food availability is specific to the St Petersburg strain. This sensitivity is likely associated with the long-term maintenance of the strain in laboratory conditions (including increased high levels of food).

Another major lesson from the hydra experiments comes from wild individuals, since this experiment in fact simulated a dietary evolutionary mismatch. Indeed, hydras in the wild consume freshwater crustaceans^55^, but not artemia which live in brackish ecosystems. Moreover, it is unlikely that a similar high-abundance and frequent diet as the one we used would be found in the wild. We therefore simulated a substantial qualitative and quantitative dietary change, and our results, showing that only individuals overfed with this new diet develop tumors, strongly supports the idea that dietary evolutionary mismatches can promote the dynamics of tumoral processes.

Finally, our experiments on hydra indicate that a high-abundance and frequent diet favors the emergence of tumors, irrespective of whether the individuals have had this diet from early in life or from five weeks of age. Thus, a lean diet early in life does not provide a lasting protective effect if a switch to food-rich lifestyles occurs later in life. This result is consistent with those of Pomatto-Watson et al.^20^ who showed that daily calorie restriction limits tumor processes better than calorie cycling. These findings support the idea that tumor processes are continuous, and that it is the availability of resources at the time they are initiated that explains whether tumors will succeed in progressing. This observation also suggests that a lean diet is protective mainly through non-genetic processes. If it was protective against mutagenic processes compared to the high-abundance and frequent diet, it would reduce or delay the appearance of tumors after the switch to food-rich lifestyle because less mutated cells would be present to fuel tumor development.

In zebrafish, the link between the emergence of tumoral processes and diet was explored in both healthy (control) individuals and individuals that are expected to develop tumors (i.e. the equivalent of the third category of hydra). In the control group, no tumors developed regardless of the diet used, while in the tumoral group there was a higher prevalence of nevi among the overfed individuals, in accordance with a previous report showing, using a different feeding system, that increased feeding enhances melanoma formation in a *p53/BRAF*-dependent model^16^. These findings confirm that a high availability of food is not in itself directly oncogenic, but it boosts the proliferation of (pre)malignant cells that are already present. Stated differently, these findings support the theoretical expectation that low resource levels are detrimental to emerging tumors^56^ because malignant cells are highly dependent on energy and/or raw material for biomass to proliferate, and/or are more sensitive to nutrient limitation and stress-induced damage. Of note, spontaneous tumorigenesis does not occur in the zebrafish non-tumoral strain regardless of the diet, whereas the hydra tumor-free strain developed tumors at a different rate depending on the diet. This result indicates a different sensitivity to diet for tumor development in these different organisms, vertebrates being less sensitive possibly due to stronger defense systems against tumorigenesis.

Results on the impact of food availability (quantity and frequency) on tumor emergence in different hydra lines and zebrafish also highlight differential sensitivities according to tumor type. Indeed, spontaneous tumors developed by hydras of the tumor-free strain, as well as those developed by zebrafish, show greater sensitivity to feeding frequency than to food quantity, in contrast to the transmissible tumors present in the hydra tumor strain. Although further research is needed to confirm this link, these preliminary results suggest that transmissible tumor cells, thanks to their longer evolutionary time than non-transmissible tumor cells, may have acquired adaptations to better withstand a momentary cut in food availability. Among other things, these adaptations could involve exploiting other metabolic pathways^57^ to ensure their maintenance while awaiting additional resources to continue to proliferate. Non-transmissible tumor cells, having just occurred in the host organism, are confronted for the first time with this selection pressure, which will result in the elimination of most of these premalignant cells. On the other hand, a low but constant food availability could be sufficient to maintain these pre-existing tumor cells, and even allow some cells to proliferate by diverting resources from the host, probably to its detriment.

### The effect of food intake on tumor progression

In hydra, we found that overfed juvenile polyps developed larger tumors relative to individuals fed lean diets. These results clearly indicate that tumor progression in tumoral polyps is positively influenced by the amount and the frequency of food intake by the host. Consistent with this finding, when the different diets were applied on older hydras with already developed tumors, overfeeding favored tumor progression while the lean diets were associated with a stabilization of tumor size, but not with regression. This supports the idea that lean diets can help control tumor size in tumor-bearing individuals, but is not curative in itself.

In zebrafish, as a measure of tumor progression we used the proportion of individuals with precancerous lesions (i.e., nevi) that developed tumors following the various diets. The overfed individuals showed higher tumor prevalence while those on lean diets were less likely to develop tumors, even if the premalignant lesions (nevi) were maintained. These data confirm that, as in hydra, high availability of food boosts the proliferation of tumor cells that are already present, and that lower resource levels help to control tumor progression but without being curative. These findings are thus consistent with the theoretical expectation that in advanced tumors, cells may have already evolved ways to survive stress such as that induced by low food availability in the tumor microenvironment^56^.

It is tempting to speculate on the reasons why the most food rich treatment – the high-abundance and frequent diet, favored tumor progression. In zebrafish, one possibility is that such high food ration can divert a considerable proportion of available energy to digestion itself, forcing an energetic trade-off resulting in less energy available to fight cancer. Indeed, high feeding rations can use up to 70% of fish aerobic scope^58^ leaving little energy for other purposes, including for fueling anti-cancer processes in the body^59,60^. Using a human analogy, this situation might be described as a ‘food coma’ akin to what we experience after eating excessively, leaving us lethargic due to energy diversion to digestion. Additional research is warranted to improve our comprehension of the phenomena operating at a mechanistic level. Various avenues could be explored, one of which is the potential generation of mutagenic reactive oxygen species, a phenomenon that might be intensified in the context of excessive food availability.

While our study draw attention to the fact that increased food intake can favor tumor development, the potential positive effects of resource availability on other aspects of fitness need also to be considered. From an evolutionary standpoint, gaining a comprehensive and holistic understanding would involve examining how overfeeding, while promoting cancer, might contribute to enhanced reproductive success in early life. This perspective is crucial for a more intricate evaluation of the selection pressures at play.

### Cancer’s vulnerability to food availability is evolutionarily conserved and reflects ancestral life history trade-offs

Our results suggest that tumor/cancer’s vulnerability to food availability is conserved among three evolutionarily distant lineages. Such deep conservation might reflect ancestral processes associated with the control of cell proliferation. Specifically, although unicellular organisms are often considered “immortal” and in a constant proliferative mode (hence the common view of cancer cells as a reversal to unicellularity; e.g.,^61^), they are able to control cell proliferation in response to signals from environment or neighboring cells. For instance, under stress (including nutrient deprivation), single-celled organisms repress proliferation to ensure long-term survival (i.e., a basic life history trade-off) (e.g.,^62^). Mutants that are unable to control proliferation do have an immediate reproductive advantage but incur long-term costs in their ability to withstand stress-induced damage (e.g., see discussion and examples in^63–70^). It has been suggested that during the transition to multicellularity, the genes involved in the expression of such trade-offs were co-opted into the evolution of the two main specialized cell types—somatic and reproductive^71,72^. Consequently, mutations in such genes can result in uncontrolled proliferation but also increased sensitivity to stress. Such antagonistic effects are thought to contribute to the stability of cooperative behaviors, including during the evolution of multicellularity, by eliminating “cheater” cells^73,74^. Trade-off reproduction for survival in response to stress can still play a role in purging cheaters—including pre-cancerous cells, in extant multicellular lineages^74^. Our findings, together with the proposed differential stress resistance of cancer cells (e.g.,^23,24^) are consistent with this possibility.

Another possibility is that reduced calorie intake reduces the incidence and progression of spontaneous tumors in both hydra and zebrafish through common mechanisms relying on evolutionary conserved intracellular pathways like the Insulin/Insulin-like growth factor (IGF) signaling pathway that connects nutrient levels to metabolism, growth, development, longevity in both vertebrates and invertebrates. Of note, *Hydra* utilizes the insulin signaling to regulate its own size^75^. This evolutionary conserved control of cell proliferation and growth promoting factors by insulin signaling might also be of particular relevance to explain the fact that cancer’s vulnerability to food availability is a conserved trait.

## Concluding remarks

Although further functional studies are needed to explain the genetic and molecular basis of our findings, this study supports the hypothesis that low food availability can have a preventive role against tumoral processes when applied at first stages of tumorigenesis, in both human and non-human systems. From an evolutionary perspective, these outcomes might reflect ancestral life history trade-offs at the cellular level that are expected to be conserved in all multicellular lineages. However, when tumoral processes are advanced, the scarcity of resources alone is not enough to be curative. On the other hand, increases in the quantity and/or the frequency of food may facilitate tumorigenesis as well as tumor progression. From an evolutionary medicine perspective, these results support the idea that fluctuations in food availability may have acted in the past (and still be acting in species in the wild) as a purging mechanism against premalignant cells that can occur spontaneously and frequently in the host body. And, furthermore, that the recent increase in food availability can by-pass this evolutionarily conserved anti-cancer mechanism and generate evolutionary mismatches leading to higher cancer risk.

### Supplementary Information

Supplementary Information 1.

Supplementary Information 2.

## Data Availability

Scripts and data are provided in supplementary information.
