# Author Correction: The impact of food availability on tumorigenesis is evolutionarily conserved

**DOI:** 10.1038/s41598-023-49920-6

**Published:** 2023-12-20

**Authors:** Sophie Tissot, Lena Guimard, Jordan Meliani, Justine Boutry, Antoine M. Dujon, Jean-Pascal Capp, Jácint Tökölyi, Peter A. Biro, Christa Beckmann, Laura Fontenille, Nam Do Khoa, Rodrigo Hamede, Benjamin Roche, Beata Ujvari, Aurora M. Nedelcu, Frédéric Thomas

**Affiliations:** 1 CREEC/MIVEGEC, Université de Montpellier, CNRS, IRD, Montpellier, France; 2 School of Life and Environmental Sciences, Deakin University, Waurn Ponds, VIC Australia; 3 Toulouse Biotechnology Institute, University of Toulouse, INSA, CNRS, INRAE, Toulouse, France; 4 MTA-DE “Momentum” Ecology, Evolution and Developmental Biology Research Group, Department of Evolutionary Zoology, University of Debrecen, Debrecen, 4032 Hungary; 5 School of Science, Western Sydney University, Hawkesbury Campus, Locked Bag 1797, Richmond, NSW 2753 Australia; 6 Hawkesbury Institute for the Environment, Western Sydney University, Penrith, NSW Australia; 7 AZELEAD, 377 Rue du Professeur Blayac, 34080 Montpellier, France; 8 School of Natural Sciences, University of Tasmania, Hobart, TAS Australia; 9 Departamento de Etología, Fauna Silvestre y Animales de Laboratorio, Facultad de Medicina Veterinaria y Zootecnia, Universidad Nacional Autónoma de México (UNAM), Mexico City, Mexico; 10 Department of Biology, University of New Brunswick, Fredericton, NB Canada

Correction to: *Scientific Reports* 10.1038/s41598-023-46896-1, published online 14 November 2023

The original version of this Article contained an error in the Funding section.

“This work was funded by the MAVA Foundation, HOFFMANN Family, and by the following grant: EVOSEXCAN project (ANR-23-CE13-0007).”

now reads:

“This work was funded by the CNRS, the MAVA Foundation, the HOFFMANN Family, and by the following grant: EVOSEXCAN project (ANR-23-CE13-0007)."

The original Article has been corrected.

