# Supplementary material for: The impact of food availability on tumorigenesis is evolutionarily conserved
Source: Sci Rep. 2023 Nov 14;13:19825. doi: 10.1038/s41598-023-46896-1 (PMC10645767; doi:10.1038/s41598-023-46896-1)
Supplement: Supplementary file 2 — Supplementary Information 2. [file 41598_2023_46896_MOESM2_ESM.docx]

**Supplementary information**

**Table S1: Hydra numbers from tumor-free and tumoral strain for each stage, batch, and diet at the start of the experiment.** The initial number of isolated individuals per group was 60, but as some individuals had died before the start of the experiment, the numbers were lower than 60, especially for the older individuals, a significant proportion of whom died before their 5-week maturity. For this category, the numbers in the last batches were deliberately unbalanced in favor of the HI and LI groups, to counterbalance the higher mortality associated with intermittent diets compared with the other two.

|  |  | High-abundance and Frequent | | High-abundance and Intermittent | | Low-abundance and Frequent | | Low-abundance and Intermittent | |
| --- | --- | --- | --- | --- | --- | --- | --- | --- | --- |
|  |  | Tumor-free | Tumoral | Tumor-free | Tumoral | Tumor-free | Tumoral | Tumor-free | Tumoral |
| Juvenile | *Batch 1* | 12 | 12 | 12 | 12 | 12 | 12 | 12 | 12 |
|  | *Batch 2* | 12 | 12 | 12 | 12 | 12 | 12 | 12 | 12 |
|  | *Batch 3* | 12 | 12 | 12 | 12 | 11 | 12 | 12 | 12 |
|  | *Batch 4* | 12 | 9 | 12 | 12 | 12 | 12 | 12 | 12 |
|  | *Batch 5* | 12 | 10 | 12 | 11 | 12 | 12 | 12 | 12 |
| Five weeks old | *Batch 1* | 12 | 12 | 12 | 12 | 12 | 12 | 12 | 12 |
|  | *Batch 2* | 12 | 12 | 12 | 12 | 12 | 12 | 12 | 12 |
|  | *Batch 3* | 12 | 5 | 12 | 6 | 12 | 5 | 12 | 5 |
|  | *Batch 4* | 7 | 3 | 12 | 3 | 7 | 3 | 12 | 4 |
|  | *Batch 5* | 12 | 3 | 11 | 12 | 12 | 6 | 11 | 10 |

**Table S2: Number of wild hydras for each diet at the start of the experiment**

| High-abundance and Frequent | High-abundance and Intermittent | Low-abundance and Frequent | Low-abundance and Intermittent |
| --- | --- | --- | --- |
| 48 | 48 | 48 | 48 |


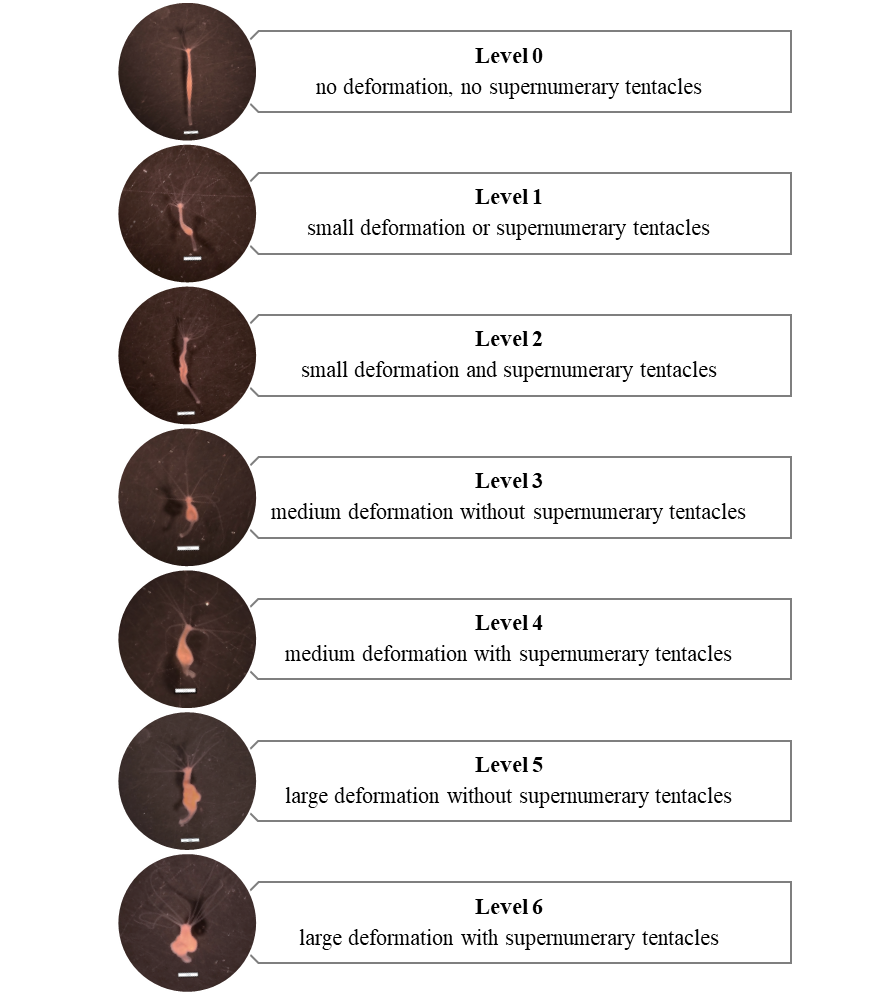


**Figure S1. Visual scale for intensity of tumor phenotype expression.** The scale used to capture the intensity of tumor phenotype expression is based on two criteria associated with the phenotype of the St Petersburg tumoral strain: size of body deformation and the presence of supernumerary tentacles *[45]*. As the tumor size is a continuous character difficult to quantify we have made a classification in 4 ordinal categories: no, small, medium, and large deformation. The justification of the link between visual tumor size and the number of tumoral cells (large GC) is presented in the Figure S2. For the tentacles, when their number equals or exceeds 8, then it is considered that the tentacles are supernumerary. The pictures were taken with a trinocular magnifier, scale bar: 1 mm.


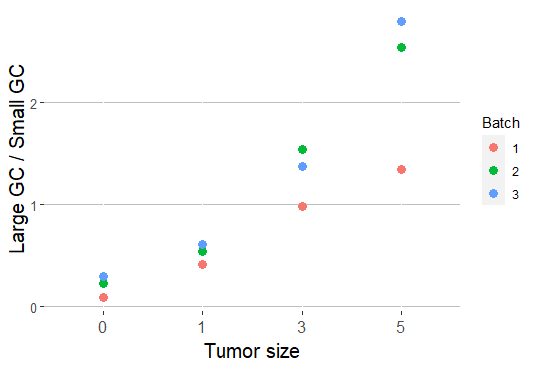


**Figure S2. Ratio of large germ cells to small germ cells of *H. oligactis* from the tumoral strain of St. Petersburg according to the visual tumor size and batch.** The macerations to obtain this result were carried out in 3 batches which are represented by different colors in the figure and taken as random effect in the analysis. The analysis reveals a positive linear relation between the ratio of large GC to small GC and the visual tumor size (taken as an ordinal ordered factor) (LMM; linear effect of the tumor size estimate = 1.53, SE =0.19, p-value < 0.001). Thus, the visual tumor size seems to be a good proxy to estimate tumor progression.

**Table S3: Composition and timing of each diet treatment for zebrafish according to their age.**

Grey squares represent dry food and beige squares the artemias; square width indict the amount of food given (small or large).

**
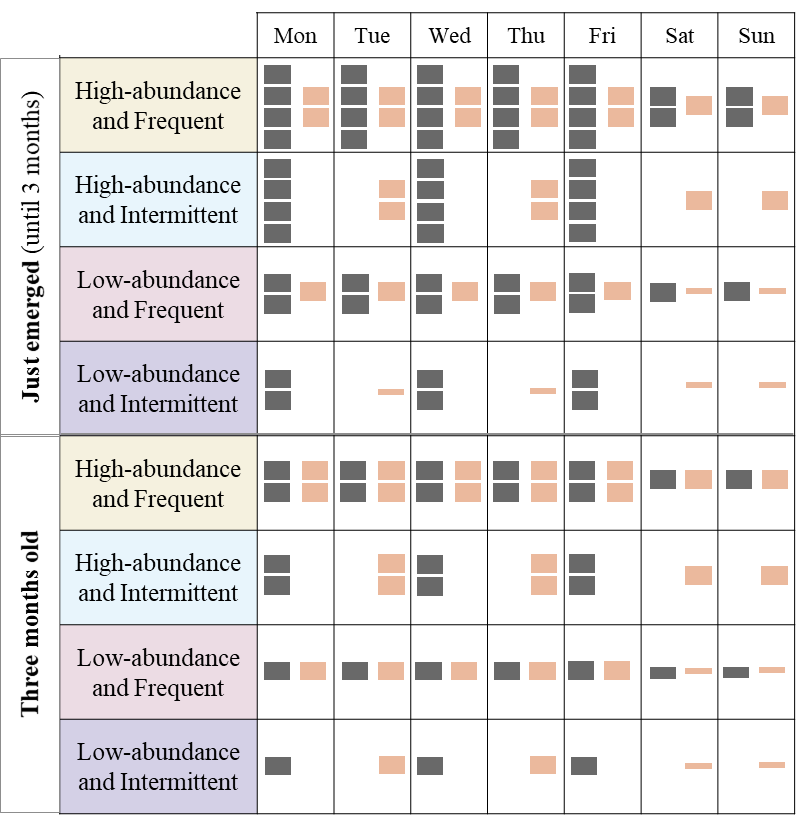
**

**Table S4: The formulas of the different models constructed and the associated value of AICc (for model with random effect) or AIC’s weight (for model without random effect), according to the biological model, the studied trait and the age**. For the analysis of the evolution of tumor size the first results are for the random effect selection and then for the fixed effects selection.

| Biological model | Model | Formulas | AIC or AICC weight | |
| --- | --- | --- | --- | --- |
|  |  |  | Juvenile individuals | Aged individuals |
| Hydras from tumor-free strain | 1.1 | Tumor development rate ~ Diet | **1** | **1** |
|  | 1.2 | Tumor development rate ~ 1 | 0 | 0 |
| Hydras from tumoral strain | 1.1 | Tumor development rate ~ Diet | 1 |  |
|  | 1.2 | Tumor development rate ~ 1 | 0 |  |
|  | 2.1 | Tumor size ~ Diet | **0.986** |  |
|  | 2.2 | Tumor size ~ 1 | 0.014 |  |
|  | 3.1 | Tumor size ~ Diet * Time + (1\|Individual) |  | **0.996** |
|  | 3.2 | Tumor size ~ Diet + Time + (1\|Individual) |  | 0.000 |
|  | 3.3 | Tumor size ~ Diet + (1\|Individual) |  | 0.001 |
|  | 3.4 | Tumor size ~ Time + (1\|Individual) |  | 0.001 |
|  | 3.5 | Tumor size ~ 1 + (1\|Individual) |  | 0.002 |
| Zebrafish from tumoral strain | 1.1 | Tumor development rate ~ Diet | **1** | **0.982** |
|  | 1.2 | Tumor development rate ~ 1 | 0 | 0.018 |
